# Effect of Group-Based Rehabilitation Combining Action Observation with Physiotherapy on Freezing of Gait in Parkinson's Disease

**DOI:** 10.1155/2018/4897276

**Published:** 2018-05-27

**Authors:** Elisa Pelosin, Roberta Barella, Cristina Bet, Elisabetta Magioncalda, Martina Putzolu, Francesca Di Biasio, Cecilia Cerulli, Mauro Casaleggio, Giovanni Abbruzzese, Laura Avanzino

**Affiliations:** ^1^Department of Neuroscience, Rehabilitation, Ophthalmology, Genetics and Maternal Child Health, University of Genoa, Genoa, Italy; ^2^Azienda Sanitaria Locale 3 Genovese, S.C. Riabilitazione Territoriale N.O. Polo Riabilitativo Levante, Genoa, Italy; ^3^Department of Experimental Medicine, Section of Human Physiology and Centro Polifunzionale di Scienze Motorie, University of Genoa, Genoa, Italy

## Abstract

Freezing of gait (FoG) is among the most disabling symptoms of Parkinson's disease (PD) patients. Recent studies showed that action observation training (AOT) with repetitive practice of the observed actions represents a strategy to induce longer-lasting effects compared with standard physiotherapy. We investigated whether AOT may improve FoG and mobility in PD, when AOT is applied in a group-based setting. Sixty-four participants with PD and FoG were assigned to the experimental (AO) or control groups and underwent a 45-minute training session, twice a week, for 5 weeks. AOT consisted in physical training combined with action observation whereas the control group executed the same physical training combined with landscape-videos observation. Outcome measures (FoG questionnaire, Timed Up and Go test, 10-meter walking test, and Berg balance scale) were evaluated before training, at the end of training, and 4 weeks later (FU-4w). Both groups showed positive changes in all outcome measures at posttraining assessment. Improvements in FoG questionnaire, Timed Up and Go test, and Berg balance scale were retained at FU-4w evaluation only in the AOT group. AOT group-based training is feasible and effective on FoG and motor performance in PD patients and may be introduced as an adjunctive option in PD rehabilitation program.

## 1. Introduction

Parkinson's disease (PD) is a neurodegenerative disease, characterized by dopaminergic and nondopaminergic degeneration, causing severe motor and nonmotor symptoms [1]. The motor manifestations of PD are manifold (tremor, rigidity, bradykinesia, and postural instability), but gait and balance disorders have a great impact on autonomy and quality of life.

Freezing of gait (FoG), defined as a “brief, episodic absence or marked reduction of forward progression of the feet despite having the intention to walk” [2], is one of the most disabling symptoms that severely impacts quality of life [3] and increases risk of falls [4] in subjects with PD. This phenomenon, commonly occurring in confined spaces or under time constraints, may be already present in the early stage of the disease and can affect up to 80% of patients in the later stages [5].

To date, both pharmacological and surgical (deep brain stimulation) treatments have been proposed to ameliorate FoG; however, the evidence of effectiveness is unsatisfying, and clear treatment protocols are still not available [6].

Regarding alternative therapeutic options (i.e. rehabilitative approaches), treatments based on behavioural strategies, requiring patients to evoke a more goal-directed type of motor control, have demonstrated to reduce freezing severity in PD patients. In this scenario, cue-augmented training surely represents one of the most effective methods for improving FoG and ameliorating gait and upper limb movements in freezers [7]. However, to date, findings are not univocal, and long-term consolidation of performance improvement needs to be investigated in future studies.

Among emerging approaches, action observation training (AOT) has been proposed as an innovative and effective method for improving gait disturbances [8, 9], bradykinesia [10], and functional independence [11] in people with PD.

Briefly, this training consists in observing and imitating specific motor actions. AOT aims to facilitate motor learning processes through the activation of the so-called “mirror neuron system” (MNS) [12]. Crucially, for the link between action observation and memory formation to be established, observed movements must be promptly executed after video observation [13].

The majority of the studies tested AOT efficacy in PD with a one-to-one (physiotherapist-patient) intervention context [14], and only one trial evaluated the feasibility of this treatment as a home-based intervention [15]. To our knowledge, no study investigated the possibility to apply AOT in a group-based rehabilitative setting. Previous data from our group have shown that AOT significantly reduced FoG episodes, producing larger and longer-lasting improvements in comparison to a control condition [8]. Interestingly, in a recent paper, clinical improvements reached after 4 weeks of AOT were also associated to functional brain changes in freezers and these changes were also able to predict clinical evolution at 8-week follow-up [9]. Based on these encouraging results and taking advantage of positive aspects related to community-based exercise programs, here, we investigated whether the AOT program may improve FoG and mobility in subjects with PD, when AOT is applied in a group-based setting. Further, by comparing the AOT program with a control program (same physical training combined with landscape-videos observation), we wanted to explore whether AOT offers beneficial effects in long-term retention of performance skills even if delivered in a group-based setting.

## 2. Methods

### 2.1. Participants

A total of 70 participants with PD were recruited at the outpatient Movement Disorders Clinic of the University of Genoa for participating in this pilot study. Patients were included in the study if they had the following inclusion criteria: (i) idiopathic PD, according to the United Kingdom Parkinson's Disease Society Brain Bank criteria [16]; (ii) Hoehn and Yahr stage II to III; (iii) able to walk unassisted despite FoG. In order to select PD subjects for those FoG severely impacted gait, patients were enrolled if the occurance of freezing was at least once a week (minimum score of 1 on item 2 in the new FoG Questionnaire (FoG-Q)) [17] and the longest episodes was > 2 seconds (minimum score of 2 on item 4 of the FoG-Q).

Participants were excluded if they had (i) diagnosis of a neurological disease (other than PD), (ii) presence of a deep brain stimulator, (iii) Mini-Mental State Examination score < 25, (iv) visual or acoustic limitations, and (v) severe orthopedic problems in the lower limbs.

Disease severity was assessed by means of section III (motor) of the Italian version of the MDS-unified Parkinson's disease rating scale (UPDRS). All patients were under treatment with dopaminergic therapy and were evaluated during the ON state (approximately 1 hour after taking their antiparkinsonian medications). Prior to any procedure, written informed consent was obtained from each participant according to our institutions' policy and was carried out in agreement with international regulations (Declaration of Helsinki, 1964) and all the subjects gave written informed consent after receiving a comprehensive explanation. Demographic and clinical characteristics of participants are reported in Table 1.

### 2.2. Training Protocol

At the end of the recruitment phase, 6 patients were excluded for not satisfying the inclusion criteria, then 64 participants were randomized to the action observation (AOT) or to the landscape observation (LOT) training by using a computerized random-number generator by an independent researcher. After the randomization process, participants were assembled in groups consisting of 5/6 patients each. Patients enrolled in the AO group-based training (*n* = 32; 14 males and 18 females; mean age ± SD: 70.4 ± 4.5) were required to carefully watch six videos (each lasting 6 minutes), in which strategies for circumventing FoG were presented and then to execute the observed actions according to the instructions given by the physiotherapist. During each group-based training session, two different video clips were presented twice and the complexity of the actions increased progressively over the sessions. Precisely, the six actions recorded in the video clips were the following: (1) shifting the body weight from one foot to the other; (2) shifting the body weight from one foot to the other making a step forward, backward and to the side; (3) walking straight with long steps; (4) turning around a chair; (5) stepping over an obstacle after shifting the body weight from one foot to the other; (6) walking through a doorway (for further details, see [8] Appendix 1). All actions shown in the video clips were performed by a physiotherapist and then projected in third-person perspective.

Participants enrolled in the LOT group (*n* = 32; 15 males and 17 females; mean age ± SD: 72.8 ± 3.1) watched six videos containing sequences of static pictures (without any human or animal representation). Precisely, during each training session LOT participants watched 2 video clips (each lasting 6 minutes and presented twice) displaying landscape scenes (e.g., pictures of mountains and seaside, countryside, and desert scenes). Then, following the physiotherapist's instructions, they performed the same actions, in the exact order and for an identical amount of time, as for the AOT group. Great care was taken to ensure that the intervention was equal across groups; thus, actions were performed following a prefixed order in both AOT and LOT groups. Therefore, the training can be considered identical for the two groups and was done by the same physiotherapist. Training sessions were scheduled 2 times per week for 5 weeks and each session lasted 45 minutes. In both group-based training, each session started with the observation of video-clips (actions or landscape images) displayed on a projector screen on the wall. To ascertain that participants focus proper attention during the video presentation, they were specifically required concentrating on what was displayed on the screen. In addition, those included in the AO group-based training were not allowed to imitate any action while observing the videos and they were not allowed to take the videos at home.

### 2.3. Outcome Measures

The objective of the study was to verify the effectiveness of group-based AOT in improving FoG and mobility. The primary outcome measure was FoG severity measured with the new FoG questionnaire. Secondary outcome measures included the effect of intervention on gait and balance performances measured by means of the Timed Up and Go (TUG), the 10-meter walking test (10 M-WT), and the Berg balance scale (BBS). Patients were always tested in their best medical condition (ON state). Assessments were performed one week before the physical therapy program (PRE), within one week after the end of the training (POST) and four weeks later (FU-4w) by an independent researcher.

## 3. Statistical Analysis

Normal distribution of data at baseline was detected by means of the Shapiro-Wilk test. Gender differences between the groups were assessed by chi-square test. Differences between groups (AOT and LOT) for age an education were assessed by the nonparametric Mann-Whitney *U* test. For UPDRS III, FoG-Q score TUG, 10 M-WT, and BBS, an unpaired *t*-test was used to compare data between groups at baseline.

To evaluate changes in the outcome measures (FoG-Q, TUG, 10 M-WT, and BBS), a repeated measure analysis of variance (RM-ANOVA) was performed with the groups (AOT and LOT) as a between-subject factor and time (PRE, POST, and FU-w4) as a within-subject factor. Post hoc analysis was performed using *t*-tests. *p* values < 0.05 were considered as a threshold for statistical significance. All statistical analyses were performed using SPSS22.

## 4. Results

All participants (*n* = 64) completed the entire cycle of group-based training; one subject from the AOT group and two participants from LOT group withdrew during the follow-up due to personal problems, and thus the adherence rate was 95.5%. At the baseline, the two groups were comparable for demographics (age, *p* = 0.31; gender, *p* = 0.61; years of education, *p* = 0.77), clinical characteristics (disease duration, *p* = 0.75; UPDRS part III, *p* = 0.88), dopaminergic daily intake (LEDD, *p* = 0.42), FoG severity (FoG-Q, *p* = 0.91), and motor performance (TUG, *p* = 0.68; 10 M-WT, *p* = 0.34; BBS, *p* = 0.33). Baseline values related to FoG severity and motor performance are reported in Table 2.

**Figure 1:**
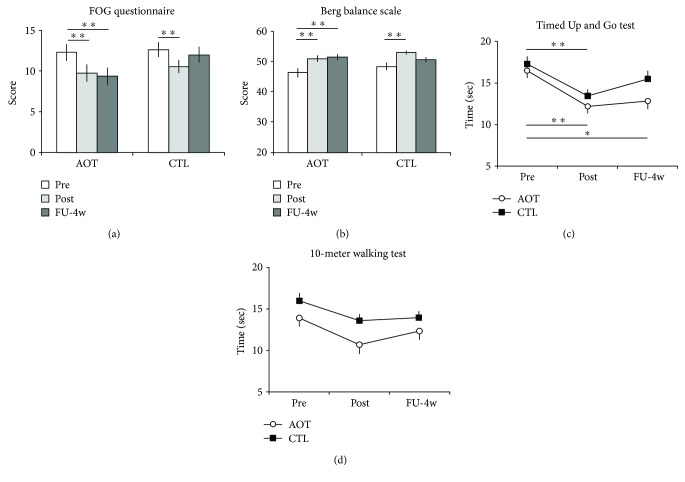
(a-b) FoG-Q and BBS mean scores (±SE). (c-d) TUG and 10 M-WT mean times (±SE). Baseline (PRE), end of training (POST), and 4-weeks follow-up (FU-4w) results are shown. Asterisks indicate when statistical significant differences within testing time evaluations emerged at post hoc analysis when the interaction term GROUP X TIME was significant (^∗^*p* < 0.05; ^∗∗^*p* < 0.001). AOT: action observation training arm; LOT: landscape observation training arm; FoG-Q: new freezing of gait questionnaire; BBS: Berg balance scale; 10 M-WT: 10-meter walking test; TUG: Timed Up and Go.

Overall results showed that both groups achieved significant improvements in all the outcome measures at posttreatment (POST) assessment, but the improvement was retained up to the FU evaluation only in AO group-based training. Indeed, statistical analysis showed a significant effect of TIME for FoG-Q score (*F*_2,59_ = 41.92, *p* < 0.001), for TUG test (*F*_2,59_ = 27.65, *p* < 0.001), and BBS (*F*_2,59_ = 17.20, *p* < 0.001) and significant group × time interactions for FoG-Q (*F*_2,59_ = 9.49, *p* < 0.001) and gait and balance performances (TUG, *F*_2,59_ = 3.52, *p* = 0.033; BBS, *F*_2,59_ = 3.24, *p* = 0.043). Further, post hoc analysis revealed that improvements in these outcome measures were retained up to the FU-4w evaluation only in the AOT group (FU-4w versus PRE: FoG-Q, *p* < 0.001; TUG, *p* < 0.001; BBS, *p* < 0.001) (Figures 1(a)–1(d)).

Differently, for the 10 M-WT data, RM-ANOVA revealed a main effect of TIME (*F*_2,59_ = 35.95, *p* < 0.001) with no significant GROUP X TIME interaction (*F*_2,59_ = 0.77, *p* = 0.46), showing that these improvements were maintained in both training arms at FU-4w (PRE versus POST, *p* < 0.01; PRE versus FU-4w, *p* < 0.01). Details of the results and statistical significances are reported in Table 2.

## 5. Discussion

The main aim of the present study was to investigate whether an AOT program delivered in a group-based setting may improve FoG and mobility in subjects with PD. Further, we wanted to explore whether AOT was more effective than standard physical therapy in long-term consolidation of rehabilitation-induced improvements, even if AOT is delivered in a group-based setting.

Here, we demonstrated, for the first time, that AOT applied in a group-based setting is feasible and more effective for long-term benefit retention than physiotherapy alone. Our results showed that the reduction of FoG severity seen after the training period in both the AOT and the control groups was maintained up to the follow-up evaluation only in PD patients trained with AOT. Indeed, improvements achieved by participants who received physiotherapy alone (LOT group) were lost after 4 weeks. Further, the long-lasting effect of AO training was seen not only for FoG severity but also for gait and balance performances.

First, we can exclude that differences in disease severity and medication intake might have exerted an impact on motor learning induced by AOT, since no differences emerged between groups related to UPDRS III score, disease duration, and Levodopa equivalent dose (LEDD).

Thus, since patients in both groups practiced exactly the same physical training and were exposed to learning the same strategies to circumvent FoG, we may postulate that the long-term retention of benefits (reduction of FoG severity at 4-weeks FU) observed only in the AOT group is likely to be related to the action observation component of training, fostering a more effective learning, possibly through a plastic effect on the MNS.

To date, a large amount of evidence has demonstrated that AOT can enhance the beneficial effects of physiotherapy by reinforcing high-level brain networks involved in motor planning and execution, by promoting imitation learning and motor control relearning [13, 18] in the elderly [19] and in patients with orthopaedic [20] and neurological diseases [21].

Regarding PD, although the number of the studies is still limited, it has been consistently showed that AOT has a beneficial effect on motor performance, especially when actions represent meaningful tasks pertaining to the patients' motor repertoire, already after a single session of AOT and to a larger extent when AOT is administered in a long-term rehabilitative program [8, 10, 11]. In line with our results, in a recent paper, it has been demonstrated that combining AO with the execution of the observed action was able to induce a positive effect on motor disability, walking speed, balance, and quality of life with a trend toward a persisting reduced freezing of gait severity up to 8 weeks after the end of the training [9] only in patients enrolled in the AOT group.

Noteworthy, these authors adopted the same video clips and identical exercises (for patients enrolled in the AOT group and control group) used in our study.

To our knowledge, only one study reported significant improvements in self-perceived mobility but no objective changes in walking performance after home-based AOT in PD [15]. Crucially, participants were not instructed to watch and repeat the observed actions although it has been reported that in AOT learning it is promoted when the task consists explicitly in “observation-execution” [13]. In addition to that, in order to achieve some benefits and foster retention effect, it seems to be fundamental teaching behavioural strategies that are suggested to shift patients' habitual motor control to a goal-directed one. Thus, the choice of actions to be displayed during AOT plays a key role in the effectiveness of this specific approach.

Freezing of gait (FoG) is a common and disabling phenomenon in PD patients [2], and it is recognized as one of the main risk factors for falls [22]. The management of FoG is complex and to date evidence is inadequate for identifying the most effective treatments [6, 7]. Developing innovative rehabilitative approaches and further reinforcing available evidence with larger studies is important for supporting both clinicians and patients in FoG management. Although the pathophysiology of FoG is still uncertain, imaging studies in PD patients with FoG pointed out the importance of cortical areas, particularly the supplementary motor area, as well as subcortical structures, including the striatum and brainstem locomotors centres [23]. A unifying idea for this network dysfunction has recently been proposed [23] suggesting a dynamic cerebral substrate for FoG. In PD patients with FoG during continuous movement (like locomotion), cortical activity in areas such as the supplementary motor area is decreased and subcortical activity is increased, perhaps to compensate the decreased cortical activity. During FoG episodes, activity in the supplementary motor area is still reduced, but subcortical hyperactivity breaks down to hypoactivity. This faulty dynamic process in cortical-subcortical activity, leading to “freezing,” might become particularly evident during challenging events that require precise regulation of step length and gait timing.

Noteworthy, a recent fMRI study [9] in PD patients with FoG showed that a 4-week AOT program was able to increase the activation of premotor cortex, inferior frontal gyrus, and left inferior parietal lobule (all areas involved in the MNS) and to influence the recruitment of frontoparietal areas that are usually involved in controlled attention and goal-directed processing in response to shifting environmental factors. Overall, these results support the idea that AOT fosters the building of motor memories, thus improving motor learning. Further, AOT is likely to interact with the faulty dynamic process involving the cortex and subcortical structures, likely through the engagement of neural circuits subserving external focus of attention. Indeed, it is well known that focused attention and external stimuli can help PD patients to overcome FoG episodes. However, this hypothesis should be confirmed in an ad hoc study testing whether AOT can interfere with cortical-subcortical activations during continuous movements in PD patients with FoG.

Finally, it is also likely that group activity facilitates adherence and stimulates the participation of subjects. Indeed, receiving exercise training in a group context may be useful for PD patients who are at higher risk of daily life and health care stigmatization [24] compared to age-matched subjects who are not suffering from the neurological disease. However, a limitation in using group-based training is that it does not allow an intervention tailored on the subjects' individual needs according to the type of freezing or other clinical features. This suggests that group-based treatment should contemplate the inclusion of patients sufficiently homogeneous for clinical features, in order to build the training program on a group's need. On the other side of the coin, our results demonstrated also that group-based training experience did not compromise the attentional capacity of participants and enabled effective learning (observational learning) of motor skills.

Some limitations of our study need to be pointed out. First, we focused on motor performance not reporting data regarding quality of life or nonmotor symptoms; second, the long-lasting effects induced by AOT were tested only 4 weeks after training; third, the screening of patients' cognitive function was limited to MMSE. Related to the latter limitation, a more comprehensive cognitive evaluation would be recommended especially in PD patients with FoG, as several studies documented a strict link between freezing and cognitive status (in particular, executive functions) [25, 26]. However, in a previous study, it has been shown that PD patients with FoG who performed worse on tests assessing visuospatial abilities, problem-solving, shifting attention, and verbal comprehension compared to controls were able to follow an AOT protocol with beneficial effects on long-term motor performance [9]. Further, it has been demonstrated that action observation triggers preserved automatic [27] and voluntary [28] imitation mechanisms even in patients with cognitive deficits due to Alzheimer's disease, particularly when patients observed a human demonstrator respect to when the stimulus was abstract. However, a recent fMRI study also showed a progressive weakening of the mirror neurons network with respect to the neurodegenerative process by comparing neural activity induced by AO in normal elderly subjects, people with amnestic mild cognitive impairment and Alzheimer's disease patients [29]. In this scenario, we think that future studies should investigate the impact of patients' cognitive profile on the immediate and long-lasting effects of AOT.

## 6. Conclusions

To conclude, our results support the efficacy of AOT, as an explicit learning process, in improving FoG in patients with PD. We demonstrated that this training is feasible and safe and can be administered even in a group-based setting thus representing an adjunctive strategy for clinicians and physiotherapists.

## Figures and Tables

**Table 1:** Demographic and clinical characteristics of participants.

	AOT	LOT	*p*
Age (years)	70.4 ± 4.5	72.8 ± 3.1	0.31
Sex (M/F)	16/17	15/16	0.61
Education (years)	9.2 ± 3.5	10.1 ± 2.2	0.77
Disease duration (years)	10.7 ± 3.9	9.5 ± 4.2	0.75
Hoehn and Yarh (stage)	2.4 ± 0.5	2.6 ± 0.3	0.73
UPDRS part III	31.6 ± 6.1	30.9 ± 7.2	0.88
LEDD (mg)	435.2 ± 158.5	383.1 ± 270.2	0.42
MMSE score	27.3 ± 2.1	28.2 ± 1.7	0.11

AOT: action observation training; LOT: landscape observation training; M: male; F: female; UPDRS: unified Parkinson's disease rating scale; LEDD: levodopa equivalent daily dose; Mg: milligrams; MMSE: Mini-Mental State Examination.

**Table 2:** Outcome measures tested before and after training and at follow-up evaluation.

Outcome measures	Action observation training group			Landscape observation training group			Repeated measure analysis of variance	
	PRE	POST	FU-4w	PRE	POST	FU-4w	Time	Time × group
FoG-Q (score)	12.3 ± 5.8	9.7 ± 5.8 ^#^	9.4 ± 5.7 ^§^	12.6 ± 5.3	10.5 ± 4.8 ^#^	12.0 ± 5.7	(*F*_2,59_ = 41.92; *p* < 0.001)	(*F*_2,59_ = 9.49; *p* < 0.001)
TUG (sec.)	16.1 ± 7.2	12.2 ± 4.9 ^#^	12.9 ± 4.1 ^§^	17.3 ± 8.1	13.4 ± 6.1 ^#^	15.5 ± 6.8	(*F*_2,59_ = 27.65; *p* < 0.001)	(*F*_2,59_ = 3.52; *p* = 0.033)
BBS (score)	46.3 ± 8.5	51.3 ± 5.7 ^#^	51.5 ± 5.5 ^§^	48.3 ± 7.1	52.4 ± 4.5 ^#^	49.6 ± 5.7	(*F*_2,59_ = 17.20; *p* < 0.001)	(*F*_2,59_ = 3.24; *p* = 0.043)
10 M-WT (sec.)	13.9 ± 4.0	10.7 ± 3.9 ^#^	12.3 ± 4.3 ^§^	15.4 ± 5.5	12.9 ± 4.3 ^#^	13.9 ± 5.4^§^	(*F*_2,59_ = 35.95; *p* < 0.001)	(*F*_2,59_ = 0.77; *p* = 0.464)

The mean ± standard deviation and details of statistical analysis are reported. FOG-Q: freezing of gait questionnaire; TUG: Timed Up and Go; BBS: Berg balance scale; 10 M-WT, 10-meter walking test; PRE: baseline evaluation; POST: after training evaluation; FU-4w: follow-up 4-weeks evaluation. Post hoc analysis: ^#^PRE versus POST and ^§^PRE versus FU-4w significant changes.
